# Acoustic CR Neuromodulation Therapy for Subjective Tonal Tinnitus: A Review of Clinical Outcomes in an Independent Audiology Practice Setting

**DOI:** 10.3389/fneur.2015.00054

**Published:** 2015-03-17

**Authors:** Mark Williams, Christian Hauptmann, Nitesh Patel

**Affiliations:** ^1^The Tinnitus Clinic Ltd., London, UK; ^2^Neurotherapies Reset NTR GmbH, Technology Center Jülich, Jülich, Germany; ^3^Section of Neuromodulation, Institute of Neuroscience and Medicine, Jülich Research Center, Jülich, Germany; ^4^Department of Otolaryngology, Whipps Cross University Hospital, Barts Health NHS Trust, London, UK

**Keywords:** tinnitus, tinnitus treatment, acoustic CR neuromodulation, neuromodulation, tinnitus therapy

## Abstract

**Objective:** To describe the quantitative treatment outcomes of patients undergoing acoustic coordinated reset (CR) neuromodulation at a single independent audiology practice over a 22- to 26-week period as part of an open label, non-randomized, non-controlled observational study.

**Methods:** Sixty-six patients with subjective tonal tinnitus were treated with acoustic CR neuromodulation with a retrospective review of patient records being performed in order to identify changes of visual analog scale (VAS, *n* = 66) and in the score of the tinnitus handicap questionnaire (THQ, *n* = 51). Patients had their tinnitus severity recorded prior to the initiation of therapy using the tinnitus handicap inventory in order to categorize patients into slight up to catastrophic impact categories. THQ and VAS for tinnitus loudness/annoyance were obtained at the patient’s initial visit, at 10–14 and 22–26 weeks.

**Results:** Visual analog scale scores were significantly improved, demonstrating a 25.8% mean reduction in tinnitus loudness and a 32% mean reduction in tinnitus annoyance with a clinically significant reduction in percept loudness and annoyance being recorded in 59.1 and 72.7% of the patient group. THQ scores were significantly improved by 19.4% after 22–26 weeks of therapy compared to baseline.

**Conclusion:** Acoustic CR neuromodulation therapy appears to be a practical and promising treatment for subjective tonal tinnitus. However, due to the lack of a control group it is difficult to reach an absolute conclusion regarding to what extent the observed effects are related directly to the acoustic CR neuromodulation therapy. Also, as the observed patient group was made up of paying clients it is unknown as to whether this could have caused any additional placebo like effects to influence the final results.

## Introduction

Tinnitus is the involuntary perception of sound, in the absence of corresponding auditory stimuli, which is perceived as originating within an individual’s ears or head. This phenomenon can affect all age groups (1), but is more likely to affect individuals who are over the age of 60 (2).

There is a strong evolving body of evidence that suggests that subjective tinnitus perception is linked to some form of peripheral audiological insult. Even subjects who have normal audiometric thresholds have been shown to have outer hair cell damage (3) and dead cochlea regions (4). This peripheral damage is thought to result in central neural plastic changes that affect the balance between excitatory and inhibitory processes leading to a potential promotion of increased afferent activity (5) and neural synchronicity (6). It is interesting to note that tinnitus generation is unlikely to be the result of an abnormally enhanced or hyperactive nerve firing pattern originating from the peripheral auditory system as sectioning of the auditory nerve does not, typically, eliminate tinnitus in pre-existing cases (7). It has also been noted that cochlea pathology causes a reduction in spontaneous firing rates in mammalian auditory nerve fibers (8). These observations serve to implicate activity changes in central structures as being the causal factor for the phantom percept generation. Cortical map reorganization has previously been suggested as being a central correlate for the emergence of tinnitus (9). However, MEG data demonstrating altered spectral power, in subjects experiencing tinnitus, suggest that this does not adequately explain the emergence of the percept in a satisfactory manner (4).

Various authors have suggested that increased auditory neural synchrony, due to a loss of inhibition, may be the cause of altered spectral power in human magnetoencephalography/electroencephalography (MEG/EEG) frequency bands. An important study investigating spontaneous brain activity in humans with tinnitus discovered an altered pattern of activity in the lower frequency EEG range with an increase of slow-wave (δ–θ) activity along with a decrease in α power within the temporal lobes (10). The perception of tinnitus intensity has actually been linked to the level of δ activity in temporal regions (11) with transient reductions in tinnitus loudness potentially being marked by concomitant reductions of δ band power (12). There is also data from studies that show auditory cortical γ band activity to be strongly increased, for noise exposed subjects, within the very early stages of tinnitus onset (13). Although it is still unclear if this elevation in γ band activity is influenced by noise-induced hearing loss. It is also important to note that studies reporting that altered spectral power, as measured by EEG/MEG, as being a neurological correlate of subjective tinnitus would benefit from being independently repeated with larger subject populations and appropriate controls.

However, it is important to note that there is a growing amount of evidence that implies that the actually salient conscious perception of tinnitus requires the involvement of a significant network of brain areas (14, 15). MEG has been used to investigate long-range cortical networks of individuals with tinnitus and demonstrated that information flow from the global network to the temporal cortex correlated positively with the strength of tinnitus distress (16). This provides evidence for the concept that percept salience is linked to an altered functional interaction between auditory and non-auditory brain areas.

There is currently no European Medicines Agency (EMA) or Food and Drug Administration (FDA) approved pharmacotherapies available for the treatment of subjective tinnitus. There is also no internationally agreed standardization relating to patient care. Treatments that are commonly utilized in order to improve quality of life include patient counseling (regarding etiology and prognosis), hearing aids, sound therapy, and cognitive behavioral therapy (17). However, with the possible exception of cognitive behavioral therapy, there is a lack of sufficiently large randomized control trials to demonstrate the efficacy of other commonly used interventions (18).

Acoustic coordinated reset (CR) neuromodulation is a non-invasive desynchronizing stimulation therapy that aims at counteracting pathological neural synchrony in subjects with subjective tonal tinnitus (6, 19). The CR algorithm has been developed computationally and is designed to desynchronize neural networks by reducing the strength of synaptic connectivity between neurones within the cell population (6, 20, 21). In order to desynchronize a synchronized focus in the tonotopically organized auditory cortex, four acoustic tones are delivered with different frequencies centered on the characteristic frequency of the participant’s tinnitus percept (19). This reduction in neural synchrony is considered to cause a decrease in the connectivity across brain areas involved in the larger salience network (15, 22). A randomized proof-of-concept trial has provided evidence for acoustic CR neuromodulation to be an effective therapy for tinnitus by demonstrating a significant improvement in visual analog scale (VAS) scores and tinnitus questionnaire (TQ) outcome measures scores for 75% of patients (19). EEG recordings for this study also demonstrated a change in pathologically altered power spectra, specifically for α, γ, and δ bandwidths, to a more normative level within a network of brain areas (19, 23) along with a significant reduction of abnormal effective connectivity (15) and cross-frequency coupling (24) within a tinnitus-related network of brain areas. It is also interesting to note that a significant number of trial participants also experienced a reduction in the characteristic frequency of their tinnitus over the course of the study (19). The results obtained in the randomized proof-of-concept trial (19) were confirmed in a real life study in 200 patients suffering from chronic tonal tinnitus (25).

This article reports the experience of a cohort of adult patients suffering from chronic subjective tonal tinnitus when treated with acoustic CR Neuromodulation over a 22- to 26-week period within an independent audiology clinic setting.

## Methods

### Subjects

**Figure 1:**
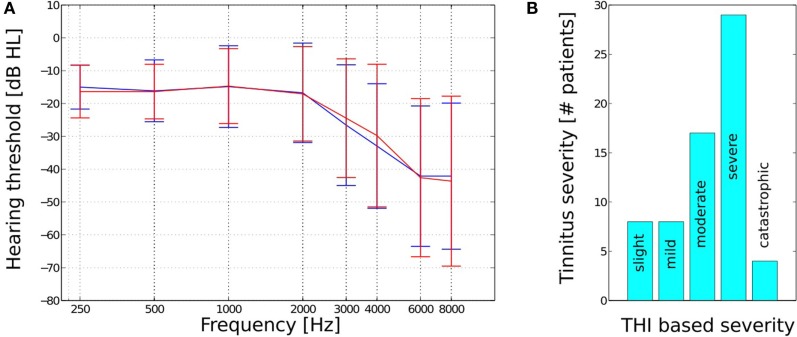
**(A)** Mean hearing thresholds and SD and **(B)** THI based tinnitus severity for the *n* = 66 subjects.

A total of 66, fee paying, participants (44 male and 22 female) ranging in age from 31 to 76 years (with a mean of 57 years and a SD of 13 years) completed 22–26 weeks of therapy. Twenty-one subjects were pre-existing hearing aid users who reported that their prescriptions improved their quality of life, 28 subjects did not have a sufficient loss to warrant the use of amplification, and 17 subjects had tried hearing aids in the past 4 years but did not report that they had benefited from their prescriptions. Pure tone audiometry revealed subjects to have sensorineural hearing losses, which ranged from mild to moderate – severe (Figure 1A). No conductive or mixed hearing losses were recorded from the patient group. All participants presented with tinnitus as their primary complaint with the length of time since tinnitus onset ranging from 3 months to 27 years (with a mean of 7 years and an SD of 8 years). Forty-two of the subjects had received tinnitus therapy previously via audiologists and psychologists in both independent and socialized healthcare clinics. The specific type of therapy utilized in these centers was diverse in nature and included diverse counseling methods, hearing aid prescriptions, tinnitus retraining therapy, sound therapies, and cognitive behavioral therapy.

The level of patient tinnitus severity was measured prior to the initiation of therapy using the tinnitus handicap inventory (THI) (26). Scoring revealed patients to be experiencing a complete range of severities from slight (0–16 points) to catastrophic (78–100 points, Figure 1B). The mean pre treatment THI score for all 66 patients was 51 (with an SD of 20). There were a total of eight patients who recorded a 0–16 point score with respect to their pre treatment THI, which is a category that has been suggested, by the THI questionnaire authors, to potentially be below the threshold of clinical significance. These low category patients did, however, report of the tinnitus percept to be sufficiently bothersome to warrant their engagement in structured therapy and determined to proceed with the intervention at their discretion.

Patients were excluded from engaging in acoustic CR neuromodulation therapy if they experienced a dominant tinnitus pitch <0.2 or >10 kHz, objective tinnitus, tinnitus that was co morbid with any acute craniomandibular or cervical–vertebral disorders, atonal tinnitus percept, had a history of Ménière’s disease, symptomatic otology disorders, any history of cervical or mandibular disorders, brainstem diseases or diagnosed psychiatric disorders, or if they were undergoing another structured treatment for tinnitus.

### Questionnaire and outcome measurement

Visual analog scale for perceived tinnitus loudness and annoyance were utilized as the primary outcome measurement. VAS loudness and VAS annoyance tinnitus metrics have demonstrated a significant convergent validity with the TQ and test–retest reliability has determined the minimum identifiable clinical difference (MICD) to be 10 points for a 100 point VAS scale (22, 27).

The tinnitus handicap questionnaire (THQ) (28) was used as the secondary outcome measurement. The THQ is a validated outcome measure, with a 0–2700 range (α = 0.95; *r* = /0.89) that is sensitive to change in tinnitus-related symptoms over time (29). Its validity, factor structure, and reliability have been independently evaluated as a sensitive metric with a test–retest reliability of 0.93 (30). THQ data from a published study of tinnitus maskers were used to estimate MICD for the metric (31) found that a difference in mean THQ score of 194 was considered significant, and represented a medium effect size. For the THQ, with a maximum score of 2700, this would equate to an individual score change of 7.1 or greater on a normalized scale (0–100) to be clinically significant. In our study, a normalized scale (0–100) was used.

Visual analog scale and THQ were recorded at the participant fitting appointment and then at their 12–14 weeks follow up appointment and finally at their 22–26 weeks follow up appointment. Prior to completing VAS and THQ, patients were required to cease active stimulation for a minimum of 15 min. Results were obtained from a total of 66 patients for VAS and 51 patients for THQ.

### Fitting and stimulation protocol

The subject’s characteristic tinnitus frequency was assessed using a manufacturer designed pure tone matching protocol, where the intensity and frequency of the matching tone were controlled by an audiologist and the patient. This method was found to be very time consuming with the approximate unilateral pitch matching time being of 45 min in duration. Pitch matching results were recorded as being repeatable to within ±5% accuracy; however, this method of therapy would benefit from utilizing a pitch matching method that can be completed in shorter period of time.

Patients were then stimulated for a 22- to 26-week treatment period using a portable acoustic device (T30 CR neurostimulator) coming with ear phones adapted from receiver-in-the-ear-canal (RIC) hearing aids that ensured that the subject’s auditory meatus was not occluded by the headphone receiver. All participants received stimulation for 4–6 h/day either continuously or divided into several sessions of a time period no shorter than one hour.

The temporal, spatial, and frequency characteristics of the generated tones were determined via the CR algorithm [Ref. (19), group 1]. This formula reflects the non-linear tonotopic organization of the auditory cortex and utilizes the matched frequency of each participant’s tinnitus percept. Acoustic CR neuromodulation employs the use of an equal number of tones that are generated above and below the subject’s specific tinnitus frequency. The stimulation tones are matched for loudness and presented at approximately 10–15 dBSPL above threshold. The presentation cycle consists of four tones played in a randomized order over three stimulation cycles followed by a pause in stimulation that lasts the equivalent time period of two presentation cycles. The cycle repetition rate was 1.5 Hz.

Patients were seen at four subsequent follow up appointments, post fitting, in order to have the characteristic pitch of their tinnitus rematched. A readjustment of the stimulation parameters could occur at these visits if the matched tinnitus frequency had changed.

### Statistical method

Descriptive statistics was used for each of the four variables (THQ, VAS loudness, VAS annoyance, and tinnitus pitch) and the three time points. A paired *t*-test was used to compare the variables to mid and end of treatment scores. The effect size was also calculated for all outcome measures using the mean and SD of the scores.

## Results

### Tinnitus loudness and annoyance

**Figure 2:**
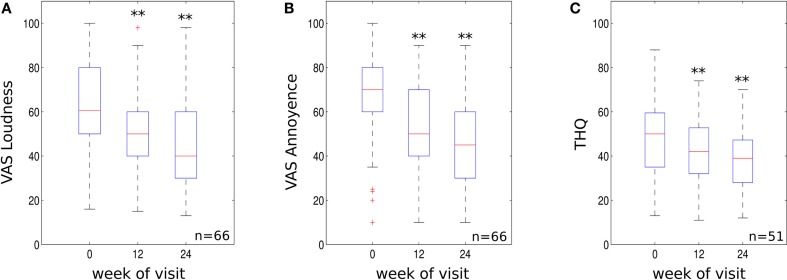
**(A)** VAS loudness scores, **(B)** VAS annoyance scores and **(C)** THQ scores for the three visits (initial visit, 10–14 weeks and 22–26 weeks visit). Data are visualized using whisker plots, significant changes are indicated by the stars (**p < 0.01).

The recording of VAS scores at follow up appointments, compared to baseline, revealed a significant reduction for both tinnitus loudness and annoyance. VAS scores for tinnitus loudness reduced by an average of 16.54 and 25.8% at the 12- to 14- and 22- to 26-week treatment mark respectively (*p* < 0.01 compared to baseline, see Figure 2A) with 59.1% of patients demonstrating a clinically significant reduction in symptoms at 22–26 weeks. Mean VAS scores for tinnitus annoyance were reduced by 21.3 and 32% at 12–14 and 22–26 weeks, respectively (*p* < 0.01 compared to baseline, Figure 2B). About 72.7% of participants experienced a clinically significant reduction for VAS annoyance after 22–26 weeks of treatment.

The effect size using mean difference and SD from base line to 22–26 weeks was 0.81 for VAS tinnitus loudness and 1.09 for VAS tinnitus annoyance. These values represent a large treatment effect.

### Tinnitus handicap questionnaire data

Tinnitus handicap questionnaire scores were significantly reduced compared to baseline. THQ scores reduced by an average of 10.3% at 12–14 weeks (*p* < 0.01 compared to baseline, Figure 2C). The clinical effect became more robust at 22–26 weeks with scores reducing by an average of 19.4% (*p* < 0.01 compared to baseline, see Figure 2C). About 58.8% of patients experienced a clinically significant reduction in THQ score at 22–26 weeks. The effect size using mean difference and SD from base line to 22–26 weeks was 0.6 for the THQ. This value represents a medium treatment effect.

### Tinnitus pitch match frequency

Sixty-four of the patients surveyed experienced a change in their pitch matched percept frequency, at 12 and 24 weeks, which required the neurostimulator system to be retuned to the newly perceived frequency. Tinnitus pitch matched frequency was reduced by 7.3% (mean reduction of 477.5 Hz) at 12–14 weeks (*p* < 0.01 compared to baseline) and by 11.1% (mean reduction of 674.3 Hz) at 22–26 weeks (*p* < 0.01 compared to baseline).

### Analysis of outcome scores for patients with slight or untroubling tinnitus

A small number of subjects within this investigation recorded a THI score of 16 points or less, which is described as being only audible in quiet environments and having no impact upon sleeping or daily activities (32). Of the seven patients who recorded a THQ at 24 weeks no beneficial clinical effect was revealed with scores remaining remarkably static at both outcome appointments (mean change at 24 weeks was 5.9%). VAS scores were recorded from the same individuals plus one additional patient at 24 weeks. Mean scores for tinnitus loudness and annoyance were shown to decrease by 9.9 and 14.0%, respectively, by 24 weeks.

## Discussion

The primary objective of this article is to report whether tinnitus sufferers could experience a reduction in tinnitus symptoms from acoustic CR neuromodulation therapy when it is delivered in an outpatient setting. The data for this small sample group demonstrate a statistically significant and clinically relevant concordant decrease of VAS scores for tinnitus loudness/annoyance and THQ scores. A similar reduction of tinnitus loudness and distress has been shown in a randomized proof-of-concept trial (19), an outpatient study (TRI conference 2011, Buffalo, NY, USA, Abstract H. Wurzer) and in a real life study in 200 patients suffering from chronic tonal tinnitus (25). The reduction of tinnitus loudness and annoyance VAS scores, within this investigation, correlated with previous work relating to tinnitus measurement variables (33). No correlation, for treatment effect, was determined with respect to patient age, gender, and audiometric configuration.

It is interesting that while patients with slight THI category scores did not report a clinically significant score change with respect to the THQ the psychoacoustic parameters of percept loudness and annoyance did decrease in a much more significant way. This may be explained by tinnitus having a very low-emotional impact on subjects in this group making the THQ metric very resistant to change even if tinnitus loudness and annoyance were decreased by the therapy.

Acoustic CR neuromodulation was well tolerated with all patients reporting, at the 24-week follow up consultation, of being able to commit to the required daily usage routine.

### Limitations to the present investigation

The clinical results that have been analyzed for this observational study do provide only very limited information on the sustainability of the therapeutic effect of acoustic CR neuromodulation, i.e., all measures were taken at least 15 min after cessation of stimulation in order to separate the sustained effects from potential masker-like effects. We know that masker-like effects usually vanish within seconds or minutes (12). We think that this relatively short observation period after stimulation offset is not long enough to name these effects long-term effects, but clear sustained effects were observable. The original proof-of-concept trial for this treatment method reported that a significant reduction in VAS scores for percept loudness/annoyance persisted at least for days and weeks after treatment cessation when patients had used acoustic CR neuromodulation for a 12-week period (19). However, the tinnitus suppression effects reduce after 4 weeks post cessation of treatment, which indicates that a continuous treatment period extending beyond 12 weeks is appropriate in order to achieve a maximal therapeutic effect. The same study also reported that TQ scores progressively improved for subjects who participated in an additional 24 weeks of therapy once the blinded trial had been completed. These results support our findings that in an outpatient setting a treatment for 36 weeks is most probably favorable as compared to a 12 weeks treatment. Treatment was provided at charge, and this could have biased findings in some unpredictable fashion. The THQ was used as an outcome measure and although this questionnaire was developed and validated with great rigor and is used widely [see Ref. (34)] other studies have deemed the third factor of this tool to not be sensitive to change (35).

This investigation would also benefit from being repeated as a clinical trial with the addition of a control group of patients who receive counseling and appropriate amplification as this would provide a direct comparison intervention with which to gage the intervention under scrutiny. This would also serve to determine what degree of improvements may have occurred as a result of a placebo effect. An extended test period to 52 weeks or beyond would provide more information on the sustainability of the intervention therapeutic effect.

It is difficult to reach a final conclusion regarding to what extent the observed effects are related directly to the acoustic CR neuromodulation therapy, since no control/placebo treatment was used in this open label, non-randomized, and non-controlled setting. However, considering that the majority of patients were shown to experience a progressive improvement in symptoms in contrast to the effect of previous structured interventions serves to make placebo induced effects improbable. A spontaneous resolution of symptom is also improbable due to the sustainable nature of the tinnitus percept in the majority patients surveyed as part of this investigation.

## Conflict of Interest Statement

Mark Williams has a contractual relationship with The Tinnitus Clinic Ltd. (UK distributor for the Acoustic CR therapy device). The results of the patient population that are reported in this manuscript were obtain from patients treated at a London based clinic owned by The Tinnitus Clinic Ltd. Christian Hauptmann is employed by Juelich Research Center; formerly working with ANM GmbH (Cologne, Germany). He works as consultant for Brook Henderson Group and has received research funding from the European Community, the Federal Ministry of Education and Research (Germany), the Deutsche Forschungsgemeinschaft, the Helmholtz Association. The Nitesh Patel declare that the research was conducted in the absence of any commercial or financial relationships that could be construed as a potential conflict of interest.
